# Temperature Regulates the Zebrafish Clock

**DOI:** 10.1371/journal.pbio.0030379

**Published:** 2005-09-27

The conveniences of modernity are not without their costs, as weary travelers know all too well. When you jet into a country halfway around the world and it's dark hours before your body expects nighttime, your internal clock doesn't have time to adjust, so you feel jet-lagged. The biological clock, calibrated to daily light and temperature cycles, controls the circadian rhythms of a wide range of physiological and behavioral processes, from fluctuating hormone levels to sleep–wake cycles and feeding patterns. While it's well known that circadian clock elements sense and respond to light cycles, evidence of temperature-dependent changes in multiple cellular processes—such as gene transcription, translation, and protein stability—in fruit flies and fungi suggest that many circadian clock components also respond to temperature cycles.

Daily temperature cycles and spikes can reset the clock's phase (timing of the peaks and troughs of activity), though its cycle length remains fixed over a wide range of temperatures. This “temperature compensation” feature confers a measure of resistance to the potentially disruptive effects of temperature fluctuations on the accuracy of the clock's timing mechanism, and appears to be a general property of the circadian system.

Since little is known about how vertebrates manage these temperature-related responses at the genetic and molecular level, Kajori Lahiri, Nicholas Foulkes, and their colleagues decided to study this question in zebrafish. This genetically tractable model organism is especially suited to this task, the authors explain, because adults, larvae, and even embryos can tolerate a wide range of core body temperatures (being cold-blooded animals) that can be manipulated simply by changing the water temperature. Temperature variations of as little as 2 °C (35.6 °F) can reset the zebrafish clock, Lahiri et al. show, and precise shifts in temperature trigger significant changes in the expression of specific clock genes.

To test whether temperature cycles can establish, or entrain, circadian rhythms in zebrafish like light–dark cycles do, Lahiri et al. raised zebrafish larvae in total darkness for six days, starting four hours after fertilization, and exposed them to a 4 °C temperature cycle. A subset of fish (at the larval stage) were sacrificed every three hours to measure RNA levels of core clock genes *(per2, per4, cry2a, cry3,* and *clock1)* and determine their expression profiles. As a control, sibling larvae were exposed to light–dark cycles and constant temperature. Clock genes *per4*, *cry2a*, *cry3*, and *clock1* showed rhythmic expression under both light–dark and temperature cycles, with the high temperature phase matching the light phase. Remarkably, the authors wrote, the results were similar for larvae raised with a daily temperature fluctuation cycle of as little as 2 °C.

Zebrafish cell lines also proved valuable tools for studying temperature response, showing a similar pattern of clock gene expression under a 4 °C temperature–darkness cycle as the larvae did under a 2 °C temperature–darkness cycle. Expression of *per4* continued even after the cells were exposed to constant temperature, an indicator of entrainment. Temperature shifts can also trigger significant changes in clock gene expression (transcript levels of *per4* and *cry3* dropped after a temperature increase and spiked after a temperature decrease; *cry2* showed the opposite response)—changes wrought by temperature-dependent shifts in the behavior of transcriptional regulators, as in the case of *per4*. Acute shifts in temperature alter the expression of several clock genes selectively. How these gene-expression responses fit into this temperature-triggered pathway is unclear, but Lahiri et al. offer a few hypotheses for future testing—investigations that should benefit from using the zebrafish cell lines the authors developed. [Fig pbio-0030379-g001]

**Figure pbio-0030379-g001:**
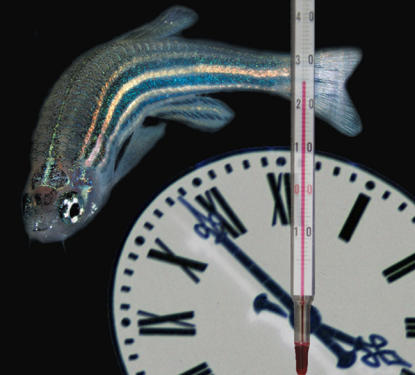
Several key transcriptional regulatory elements at the core of the zebrafish circadian clock respond to temperature

The authors go on to show that the zebrafish clock functions over a range of temperatures with characteristic temperature compensation. They speculate that this may result when temperature changes produce shifts in the amplitude of circadian transcription rhythms. Altogether these results show that temperature can regulate the circadian clock in this vertebrate. If the temperature-induced transcriptional responses described here operate in other temperature-related responses, they may shed light on how temperature affects other biological systems as well. —*Liza Gross*

